# Prevalence and Pattern of Congenital Heart Disease Among Children in Khartoum State, Sudan: A Reflection of the Current Cardiac Profile

**DOI:** 10.7759/cureus.21196

**Published:** 2022-01-13

**Authors:** Osman Abdelrahman, Randa Diab

**Affiliations:** 1 Anatomy, University of Medical Sciences and Technology, Khartoum, SDN; 2 Anatomy, Ahfad University for Women / National University - Sudan, Khartoum, SDN

**Keywords:** cardiology, sudan, pattern, prevalence, congenital heart disease

## Abstract

Introduction

Congenital heart diseases (CHDs) are common anomalies that increase morbidity and mortality among children and adolescents. It impacts the lifestyle of survivors who live with one defect of the minor spectrum of CHD. Our research goal was directed to estimate the prevalence of CHD in Khartoum State, Sudan. Furthermore, we compared the pattern of CHD we acquired with that determined in a previous study in our country during the early nineties of the twentieth century (1994).

Methods

This is an observational cross-sectional study done by reviewing the medical records of 596 patients diagnosed with CHD from pediatric hospitals in Khartoum State between January 1, 2018, to December 31, 2019. We used a checklist with closed-ended statements; this was reviewed by pediatric cardiologists.

Results

Prevalence was determined to be 14.3/1000. There was a male predominance of 56.7%. Ventricular septal defect (VSD) was the most commonly occurring lesion (26.6%), followed by tetralogy of Fallot (TOF; 14.1%) and then patent ductus arteriosus (PDA; 10.6%). The most common combined anomalies were transposition of great arteries (TGA) and patent foramen ovale (PFO) (9.1%).

Conclusion

The prevalence of CHD in Khartoum State is estimated to be 14.3/1000, which is nearly similar to some countries in Africa but higher than most of the continent's countries. VSD was the most common isolated anomaly among CHD patients. There was slight male predominance. Our findings could be used to plan appropriate long-term strategies; to prevent a further rise in the prevalence of CHD. In addition, to be capable of investigating the predisposing factors behind CHD.

## Introduction

Congenital heart disease (CHD) is a defect or group of defects that occur at the level of the heart anatomy, affecting its function, which can be detected during varied ages from the beginning of life or later on [1]. CHD worryingly puts considerable weight on the morbidity and mortality of infants globally [2]. Even from an economic point of view, it consumes enormous resources in terms of surgical repair procedures and costs for hospital stays to recover from surgical procedures [3].

CHD is the most occurring anomaly when compared to the rest of fetal abnormalities in developed countries [4-8]. In some parts of the world, the estimation of deaths attributed to CHD was greater than deaths that occurred due to malignancies [5]. Despite the awareness of the CHD burden on the healthcare system, its etiology is still not well understood. The etiology is believed to be multifactorial, including genetic and environmental factors [4].

This study was conducted to estimate the prevalence of CHD among children in Khartoum State and to update the pattern of CHD. Also, one of the main goals of this study was to develop a database of CHD in Khartoum State, as there is no available data to the best of our knowledge. Our study adds facts regarding CHD, which can be used in the future to plan clear strategies regarding public awareness about CHD. It will also open the door for policymakers to make the right decisions. Hence, investigating and assessing factors behind CHD and monitoring any rise or drop at the level of prevalence in the future would be applicable.

## Materials and methods

Study design and sample size

A descriptive cross-sectional study design was applied to estimate the prevalence of CHD in Khartoum State, Sudan, in the period from January 2018 to December 2019. Khartoum State has four major pediatric hospitals, Ahmed Gasim Pediatric Hospital, Jafar Ibn Ouf Pediatric Hospital, Omdurman Pediatric Hospital, and Sudan Heart Center, distributed within three cities within Khartoum State - Bahri, Khartoum, and Omdurman.

All medical records of children diagnosed with CHD were covered. The period of the study was from January 1, 2018, to December 31, 2019. Five hundred ninety-six CHD patients came to the hospitals mentioned during this period. Data were collected from their medical records using a pretested checklist with closed-end statements and reviewed by three pediatric cardiologists.

The independent variables in this study were a family history of similar conditions, maternal lifestyle, and maternal drug history. Moreover, maternal illnesses during pregnancy, maternal history of chronic illnesses, maternal age during pregnancy with the affected child, maternal antenatal visits, and the use of folic acid supplementation during pregnancy, were considered independent variables as well.

Data analysis

The data were entered and processed using Microsoft Excel version 2013 (Microsoft Corporation, Redmond, WA). Analysis of data by obtaining descriptive frequency tables and graphs using the Statistical Package for Social Sciences (SPSS) version 23 software computer package (IBM Corp., Armonk, NY).

Ethical considerations

The research proposal was approved by the research committee of the National University - Sudan. Accordingly, letters to hospital administrations were included in this study and prepared and stamped by the university. In the final step, permission was obtained from the administration of each hospital to collect data from medical records. There was no contact with patients during the study.

## Results

After reviewing 596 records found in Khartoum State pediatric hospitals, in the period between January 1, 2018, and December 31, 2019, the total prevalence of CHD was calculated to be 14.3/1000.

The prevalence was calculated by dividing the total number of cases of CHD by the total number of pediatric cases (cardiac and non-cardiac) that came to the hospitals during this period. Before the total prevalence was calculated, each year's prevalence was calculated separately. The total number of CHD cases in 2018 was 279, and the total number of pediatric cases (cardiac and non-cardiac) was 20,041 cases. The total number of CHD cases in 2019 was 317, and the total number of pediatric cases (cardiac and non-cardiac) was 21,396.

An increase in the prevalence from 2018 to 2019 was noticed in this study. The prevalence of 2018 was measured to be 13.9/1000 while the prevalence of CHD in 2019 was measured to be 14.8/1000 (Table 1).

**Table 1:** Prevalence of congenital heart disease among children in Khartoum State, Sudan, 2018-2019

Year	Total number of cases	Total number of pediatric cases	Prevalence
2018	20,041	279	13.9/1000
2019	21,396	317	14.8/1000
Overall	41,437	596	14.3/1000

The age of children affected with CHD at the time of diagnosis was studied, and the majority of children were less than two years of age (67.1%) (Table 2).

**Table 2:** Age of children at the time of congenital heart disease (CHD) diagnosis in Khartoum State, Sudan, 2018-2019

Age	Frequency	Percent
Less than 2 years	400	67.1%
2-5 years	81	13.6 %
6-9 years	58	9.7%
10-13 years	37	6.2%
14-17 years	20	3.4%
Total	596	100%

There was male predominance when the gender distribution among CHD children was studied; 56.7% of the CHD patients were males while 43.3% were females (Table 3).

**Table 3:** Sex distribution of children diagnosed by congenital heart disease (CHD) in Khartoum State, Sudan, 2018-2019

Gender	Frequency	Percent
Female	258	43.3%
Male	338	56.7%
Total	596	100%

Moreover, consanguinity between parents of children affected with CHD was addressed in this research, and the majority of parents were non-consanguineous (73.5%). Family history of CHD was negative in the majority of cases (97%). In terms of geographical distribution within our country, most of the children affected with CHD were from central states (37.9%), followed by those from western states (25.8%), whereas the children affected with CHD who are from the southern states were the lowest in number. Ninety-six percent (96%) of mothers of children diagnosed with CHD were housewives.

Regarding the pattern of CHD, the study showed that the most prevalent single CHD was ventricular septal defect (VSD) (26.6%). The next most common anomaly was tetralogy of Fallot (TOF) (14.1%) while patent ductus arteriosus (PDA) was the third most common anomaly (10.6%). The least single CHD was the bicuspid aortic valve (0.5%). Considering the multiple defects, transposition of great arteries (TGA) and patent foramen ovale (PFO) occurring together were seen in 9.1% of the CHD cases, followed by VSD and pulmonary stenosis (PS), which accounted for 3.9%. The least combined CHD were PDA and VSD, which accounted for 0.3% (Table 4).

**Table 4:** The pattern of congenital heart disease among children in Khartoum State, Sudan, 2018-2019

Type of CHDs	Frequency	Percentage
Ventricular septal defect (VSD)	159	26.6%
Tetralogy of Fallot (TOF)	84	14.1%
Patent ductus arteriosus (PDA)	63	10.6%
Transposition of great arteries (TGA) + patent foramen ovale (PFO)	54	9.1%
Atrial septal defect (ASD)	37	6.2%
Transposition of great arteries (TGA)	35	5.9%
Ventricular septal defect (VSD) + pulmonary stenosis (PS)	23	3.9%
PS	22	3.7%
Atrioventricular septal defect (AVSD) + Down syndrome (DS)	20	3.4%
Tricuspid atresia (TA)	16	2.7%
AVSD	12	2.0%
Total anomalous pulmonary venous drainage (TAPVD)	10	1.7%
Ebstein anomaly	7	1.2%
Aortic stenosis (AS)	7	1.2%
ASD + PS	6	1.0%
PS + AVSD	6	1.0%
VSD + dextrocardia	6	1.0%
Coarctation of aorta (COA)	6	1.0%
Dextrocardia	5	0.8%
Truncus arteriosus (TrA)	5	0.8%
TrA + VSD	5	0.8%
TGA + dextrocardia	3	0.5%
Bicuspid aortic valve	3	0.5%
PDA + VSD	2	0.3%
Total	596	100%

## Discussion

To the best of our knowledge, it is the first study of its type to address the measurement of the prevalence of CHD in the Khartoum State, Sudan. The prevalence of CHD was estimated as 13.9/1000 for 2018 and 14.8/1000 for the year 2019. As it appears, the prevalence of CHD estimated in 2018 was lower than that measured in 2019. This observation might be attributed to a deterioration in the preventive strategies offered by health providers in addition to the lack of awareness enhancement campaigns directed toward CHD, targeting women of childbearing age.

The overall prevalence of two years was 14.3/1000. When we look at the numbers regarding CHD in Africa, we consider our estimated prevalence to be almost similar to the prevalence of CHD measured in Nigeria (14.4/1000) [5]. Meanwhile, it was considered higher than the prevalence estimated in Senegal, Mozambique, and Kenya (8.9, 2.3, and 1.8 per 1000, respectively) [9-11]. Our results were close to some countries in Africa because we share the same economic status, environment, political conflicts, and health services. However, the low prevalence measured in Mozambique and Kenya should not be compared with ours, as theirs was measured 15 and 25 years ago, respectively; moreover, diagnostic methods and the population were hugely less than nowadays.

Our results are lower than the prevalence measured in Asian countries such as India and China (19.4 and 22.9 per 1000, respectively) [6,12]. But it was higher than the prevalences in Iran and Pakistan (8.6 and 3.4 per 1000, respectively) [4,13].

When we compared our results to those from Australia, Europe, and North America, we noticed that our prevalence is higher. It would be clear when we look at the lower prevalences of Denmark, Australia, the USA, and Turkey (6.1, 7.65, 3.7, and 7.77 per 1000, respectively) [14-17]. The difference could be attributed to poor economic status, lack of awareness, poor education, and poor health services in our country. We might underestimate our prevalence because almost 20% of children in this study were diagnosed with CHD after the age of five years, so many patients may not appear to the medical attention yet.

Regarding the pattern of CHD noticed in this study, VSD was the most occurring anomaly (26.6%), followed by TOF (14.1%) and then PDA (10.6%). These anomalies accounted for 51.3% of the total CHD patients records included in our study. This finding displays that our pattern is changing, as it was determined in our country in 1994 to show that VSD was the most common lesion followed by TOF and then PS [18].

We do share similarities with the rest of the world in the fact that VSD is the most occurring anomaly among CHD, starting from African countries like Ethiopia and Nigeria [19-20] to Asian countries like India and China [21-22], as well as developed countries like Denmark, Turkey, and Czech [14,17,23].

Sex distribution among CHD traced, and there was male predominance (56.7%). Females accounted for 43.3%. Awareness of the mothers about CHD seems to justify our high prevalence, as 96% of them are housewives with medium to low levels of education. The majority of CHD patients were born to non-consanguineous parents (73.5%), and the majority of them had negative family histories of CHD (97%). However, linking CHD with a family history of heart diseases and consanguinity cannot be assessed, as no autopsies have been done for children who died suddenly in their families.

Regarding the origin of CHD patients, the majority were originally from central states followed by western states. Hence, possible environmental factors could be investigated in such states besides genetic analysis in the future.

By knowing the previously mentioned factors regarding the CHD profile in our country, we can build new strategies based on actual figures to investigate the factors contributing to CHD occurrence. In addition, we can monitor the prevalence and pattern in the following years, as we have reference numbers now.

We looked for multiple risk factors like maternal age, maternal chronic illnesses, maternal febrile illnesses during pregnancy, antenatal care visits, folic acid supplementation, smoking, alcohol consumption, illicit drugs, and medication taken during pregnancy. Unfortunately, due to the poor quality of records reviewed, there was a lot of missing data that made us unable to detect the previously mentioned risk factors. We did find risk factors in a few numbers of the records. However, it will never be representative. It was our main obstacle and limitation in this study.

I recommend improving the quality of records in hospitals by making clear standards and instructions to follow during the process of filling the medical records, which should be double-checked by the quality department upon patient discharge. Moreover, I suggest putting the records of the patients in a computer system to facilitate their retrieval as needed. Such steps can help us investigate the possible risk factors contributing to CHD, as we still have no idea about it in our country. Finally, I suggest conducting a series of researches, both prospective and retrospective, to add the missing information in this field.

## Conclusions

This cross-sectional study conducted in Khartoum State yielded a prevalence of CHD of 14.3 per thousand individuals, when measured during January 2018 and December 2019. The prevalence of CHD for the year 2019 was higher than the CHD prevalence of 2018, the former being 13.9 per thousand with the latter being 14.8 per thousand. Concerning the pattern of CHD, VSD was the most commonly occurring isolated lesion, followed by TOF and then PDA. TGA with PFO has been determined as the most commonly occurring combined cardiac lesions. According to our study, more males were affected by CHD than females. An explanation for such an observation was not detected during the study. The findings of this study can be applied to get plans for both the clinical and public health sectors.
